# Salbutamol and around

**DOI:** 10.1186/1824-7288-41-S2-A74

**Published:** 2015-09-30

**Authors:** N Ullmann, S Caggiano, R Cutrera

**Affiliations:** 1 Respiratory Unit, Bambino Gesù Paediatric Hospital, IRCCS, Rome, Italy; 2 Sapienza University, Rome, Italy

## 

Short-acting beta agonists (SABAs) such as salbutamol are well known as the first-line therapy for the treatment of acute exacerbations, exercise-induced asthma and childhood intermittent asthma. As needed, SABAs with no controller should be considered only if symptoms are rare and there is no awakening due to asthma. Salbutamol, through its effects of relaxing airway smooth muscle and increasing airflow, provides rapid relief of acute asthma symptoms. Treatment's effects begin in about 10-15 minutes and peak effect is usually within 30 minutes. Patient experience prompt benefit with reduction in coughing, wheezing, chest tightness and shortness of breath. The treatment dose includes 4-10 puffs by pMDI+spacer every 20 minutes for 1 hour [1]. The rapid relief provided by salbutamol may invite misuse and overuse, particularly in adolescents. SABAs should not be prescribed on a regular schedule as it may lead to a beta receptor downregulation. Moreover, frequent use of salbutamol is an indication of poor asthma control and an increased use of controller therapy should be considered. An heterogenous response to beta agonist therapy has been described and individual response is related to many factors, including: the relative contribution of bronchoconstriction versus airway inflammation and edema in producing the airway obstruction, different triggering mechanisms, the duration of symptoms, the patient's age and the route of medication delivery. Salbutamol is normally delivered through the inhaled route while intravenous salbutamol is used when children are unresponsive to inhaled treatment [A]. Less common are the oral and subcutaneous routes. Inhaled SABAs, administered through a MDI with attached spacer device along with infant- or child-sized mask or by nebulization, have the advantages of smaller doses and fewer side effects [3]. Inhaled SABAs allow selectivity for beta-2 receptors on bronchial smooth muscle to achieve bronchodilation without significant tachycardia associated with activation of beta-1 receptors on cardiac muscle. The incidence of adverse effects is dependent upon age of patient, dose, and route of administration. Examples of possible mild or moderate side effects in children and adolescents, not very common at recommended doses, include: palpitations, tachycardia, excitement, hyperactivity, insomnia, nervousness, shakiness and unpleasant taste (inhalation site) [4].
